# Childhood trauma is associated with reduced frontal gray matter volume: a large transdiagnostic structural MRI study

**DOI:** 10.1017/S0033291721002087

**Published:** 2023-02

**Authors:** Marieke J. H. Begemann, Maya J. L. Schutte, Edwin van Dellen, Lucija Abramovic, Marco P. Boks, Neeltje E. M. van Haren, Rene C. W. Mandl, Christiaan H. Vinkers, Marc M. Bohlken, Iris E. C. Sommer

**Affiliations:** 1 Department of Biomedical Sciences of Cells & Systems, Section Cognitive Neurosciences, University Medical Center Groningen, University of Groningen, Groningen, the Netherlands; 2 Department of Psychiatry, UMCU Brain Center, Utrecht University, Utrecht, the Netherlands; 3 Department of Child and Adolescent Psychiatry/Psychology, Erasmus University Medical Center-Sophia Children's Hospital, Rotterdam, the Netherlands; 4 Department of Psychiatry, Amsterdam UMC (location VUmc), Amsterdam, the Netherlands; 5 Department of Anatomy and Neurosciences, Amsterdam UMC (location VUmc), Amsterdam, the Netherlands

**Keywords:** Bipolar disorder, childhood trauma, frontal lobe, gray matter volume, psychosis

## Abstract

**Background:**

Childhood trauma increases risk for psychopathology and cognitive impairment. Prior research mainly focused on the hippocampus and amygdala in single diagnostic categories. However, other brain regions may be impacted by trauma as well, and effects may be independent of diagnosis. This cross-sectional study investigated cortical and subcortical gray matter volume in relation to childhood trauma severity.

**Methods:**

We included 554 participants: 250 bipolar-I patients, 84 schizophrenia-spectrum patients and 220 healthy individuals without a psychiatric history. Participants filled in the Childhood Trauma Questionnaire. Anatomical T1 MRI scans were acquired at 3T, regional brain morphology was assessed using Freesurfer.

**Results:**

In the total sample, trauma-related gray matter reductions were found in the frontal lobe (*β* = −0.049, *p* = 0.008; *q* = 0.048), this effect was driven by the right medial orbitofrontal, paracentral, superior frontal regions and the left precentral region. No trauma-related volume reductions were observed in any other (sub)cortical lobes nor the hippocampus or amygdala, trauma-by-group (i.e. both patient groups and healthy subjects) interaction effects were absent. A categorical approach confirmed a pattern of more pronounced frontal gray matter reductions in individuals reporting multiple forms of trauma and across quartiles of cumulative trauma scores. Similar dose−response patterns were revealed within the bipolar and healthy subgroups, but did not reach significance in schizophrenia-spectrum patients.

**Conclusions:**

Findings show that childhood trauma is linked to frontal gray matter reductions, independent of psychiatric morbidity. Our results indicate that childhood trauma importantly contributes to the neurobiological changes commonly observed across psychiatric disorders. Frontal volume alterations may underpin affective and cognitive disturbances observed in trauma-exposed individuals.

## Introduction

Exposure to childhood trauma, encompassing different forms of maltreatment early in life, is an important risk factor for most psychiatric disorders (Green et al., 2010; Varese et al., 2012). Childhood trauma may affect brain development, leading to aberrant cognitive functioning and emotional regulation and subsequent behavioral dysfunction (Lupien, McEwen, Gunnar, & Heim, 2009; Sánchez, Ladd, & Plotsky, 2001; Teicher & Samson, 2016). Patients with a history of trauma may therefore form a specific group in terms of symptom presentation, response to treatment and clinical needs. As this subgroup is fairly large, roughly varying between 40% and 60% across psychiatric disorders, identification of trauma-related features of psychopathology, including their underlying neurobiological correlates, may inform individually tailored mental health care (Álvarez et al., 2011; Devi et al., 2019; Green et al., 2010; Porter, Branitsky, Mansell, & Warwick, 2020; Schäfer & Fisher, 2011; Wiersma et al., 2009).

Evidence on trauma-related gray matter alterations is now accumulating from structural magnetic resonance imaging (sMRI) investigations. Prior literature has largely discussed the effect of childhood trauma on the hippocampus and amygdala (Lim, Radua, & Rubia, 2014; Paquola, Bennett, & Lagopoulos, 2016). This primary focus was guided by early findings in animal studies on the effect of early life stress impacting these regions, and clinical observations that linked childhood trauma to reduced memory performance and anxiety/mood regulation (Myers, McKlveen, & Herman, 2014). Early MRI studies investigating the effect of trauma in patient samples have often used a region-of-interest (ROI) approach with hippocampus and amygdala as only ROIs (Lim et al., 2014; Paquola et al., 2016), overlooking the role of trauma in other areas. The gray matter correlates of childhood trauma may well extend beyond the hippocampus and amygdala, as suggested by (pre)clinical childhood trauma models that implicate more widespread corticostriatal-limbic involvement (Edmiston et al., 2011; Monroy, Hernández-Torres, & Flores, 2010). Clinical studies also indicate a more extensive negative impact of childhood maltreatment involving a wide range of affective and cognitive functions, such as reward processing, attention and executive functioning, which are not primarily supported by hippocampus and amygdala function (Hart & Rubia, 2012; Pechtel & Pizzagalli, 2011). Such complex, higher-order functions are rather represented by networks that encompass both subcortical and cortical brain structures (Menon, 2011). Thus, childhood trauma is hypothesized to lead to broader gray matter volume reductions in distributed cortical and subcortical areas, which is thought to impact cognition and emotion regulation and could be an independent risk factor for the development of psychopathology.

Despite the hypothesized general impact of childhood trauma, most of our knowledge on its neurobiological correlates stems from studies investigating individual diagnostic categories, including post-traumatic stress disorder, mood and anxiety disorders such as bipolar-I disorder, and schizophrenia (Teicher & Samson, 2016). As childhood trauma may affect patients with different diagnoses in a similar vein prior to the development of psychopathology, it is important to investigate its neurobiological consequences using a transdiagnostic approach (Bennett & Lagopoulos, 2018; Paquola et al., 2016). Moreover, as only a minority of maltreated children will develop a psychiatric illness later in life, assessing the impact of childhood trauma both in the presence *and* absence of psychopathology will help disentangle the neurobiological effects of trauma from diagnostic classification (Begemann, Schutte, & Sommer, 2015b; Bennett & Lagopoulos, 2018; Paquola et al., 2016).

In this study, we aimed to identify trauma-related gray matter alterations across the brain by investigating both cortical and subcortical regions in an exploratory manner. Moreover, we aimed to find correlates of trauma across diagnostic boundaries, by analyzing patients with disorders in two distinct DSM-V categories that share childhood adversity as an environmentally contributing factor. Relative to the general population, trauma-exposed individuals have a three-fold increased risk of developing bipolar disorder (Palmier-Claus, Berry, Bucci, Mansell, & Varese, 2016) or psychosis (Varese et al., 2012). Therefore, we included 250 bipolar-I disorder patients and 84 patients with a schizophrenia-spectrum disorder. We also included a sample of healthy individuals (*n* = 220) to investigate possible correlates of trauma resilience.

## Methods

### Participants

Data were obtained from MRI studies conducted in the University Medical Center Utrecht (the Netherlands): *Bipolar Genetics study* (Vreeker et al., 2016), *Spectrum study* (Sommer et al., 2010) and *Simvastatin for recent-onset psychosis* (baseline data) (Begemann et al., 2015a). Participants were ⩾18 years of age. We included 554 participants: 220 healthy individuals without a current or past psychiatric diagnosis, 251 patients with type-I bipolar disorder, and 83 patients with a schizophrenia-spectrum disorder (DSM-IV-TR) (American Psychiatric Association, 2000). Presence or absence of psychopathology was established using the Structured Clinical Interview for DSM-IV (SCID-I) (First, 1997), the Mini-International Neuropsychiatric Interview (Sheehan et al., 1998) or the Comprehensive Assessment of Symptoms and History Interview (CASH) (Andreasen, Flaum, & Arndt, 1992). Studies were approved by the medical ethical committee, participants gave written informed consent. Procedures are described in more detail in the eAppendix in Supplement.

### Childhood trauma

Childhood maltreatment was assessed with the Dutch version of the Childhood Trauma Questionnaire–Short Form (CTQ-SF total score) (Bernstein et al., 2003; Thombs, Bernstein, Lobbestael, & Arntz, 2009). This 25-item version evaluates five trauma subtypes (sexual, physical and emotional abuse, physical and emotional neglect), summing up to a total score. Good internal consistency reliability has been reported in both clinical and community samples (Bernstein et al., 2003; Scher, Stein, Asmundson, McCreary, & Forde, 2001). Established cut-off scores for moderate to severe trauma were applied to classify the presence of a specific trauma subtype (emotional neglect ⩾15; physical neglect ⩾10; emotional abuse ⩾13; physical abuse ⩾10; sexual abuse ⩾8) (Bernstein et al., 2003).

### Structural magnetic resonance imaging

Structural MRI scans of the whole brain were acquired on the same 3T Philips Achieva medical scanner, equipped with an 8-channel SENSE headcoil (Philips, The Netherlands). Three-dimensional high-resolution T1-weighted images (high-res T1) were obtained with the following parameters; 200 contiguous sagittal slices (TE = 4.6 ms, TR = 10 ms, flip angle = 8°, FOV = 240 mm,0.75 × 0.75 × 0.80 mm^3^ voxels). T1-weighted images (T1) with a slightly lower resolution (160 contiguous sagittal slices, TE = 4.6 ms, TR = 10 ms, flip angle = 8°, FOV = 224 mm, 1 × 1 × 1 mm^3^ voxels) were available for 43 healthy participants and 33 schizophrenia-spectrum disorder patients, yet no differences in total gray matter volume were observed when contrasting individuals with lower *v.* higher resolution T1 images within these groups (*p* = 0.651 in the healthy control group; *p* = 0.323 in the schizophrenia-spectrum group).

All 554 scans were checked for radiological abnormalities by a radiologist blinded to subject status (healthy individual or patient). Structural images were processed using the automated segmentation pipeline of FreeSurfer version 5.3.0 (http://surfer.nmr.mgh.harvard.edu) (Fischl et al., 2004). By automatic parcellation of the cortical and subcortical structures, gray matter was divided into 82 distinct anatomical volumes of the Desikan−Killiany atlas (Desikan et al., 2006). Segmentations were manually checked for volumes deviating significantly from the population mean [i.e. more than two standard deviations (s.d.)] regarding total gray matter volume, thickness (left and right), surface area (left and right) and cerebral brain volume. When visual inspection of these images showed gross scan or segmentation errors, these segmentations were deemed unfit for further analysis. Images from two participants showed radiological abnormalities, two scans showed segmentation faults and seven scans were of insufficient quality, resulting in 543 scans suitable for analyses. The eleven excluded participants (four healthy controls, two bipolar-I patients and five schizophrenia-spectrum disorder patients) did not significantly differ (>2 s.d.) from the remaining sample across demographic variables or childhood trauma score. For each participant, total gray and white matter volumes were combined to calculate a general measure of cerebral brain volume.

We hierarchically grouped brain regions in order to obtain sufficient statistical power for analyzing both cortical and subcortical areas. The 68 individual cortical regions first were grouped into five cortical lobes: frontal (*n* = 22), parietal (*n* = 10), occipital (*n* = 8), temporal (*n* = 18) and insula/cingulate (*n* = 10) (Allen, Damasio, & Grabowski, 2002; Allen, Damasio, Grabowski, Bruss, & Zhang, 2003; Collin et al., 2013; Desikan et al., 2006; Long et al., 2012). The 14 subcortical regions were grouped into one subcortical lobe, including the bilateral nucleus accumbens, amygdala, caudate, hippocampus, pallidum, putamen and thalamus. For significant lobes, individual regions were evaluated to investigate their contribution to the effect.

### Statistical analysis

Statistical analyses were performed using SPSS software, version 22.0. Group-differences in demographic characteristics were evaluated using chi-squared tests for categorical variables and *F*-tests for continuous variables. Differences in total cerebral brain volume were evaluated using ANCOVA (age and sex as covariates). Significant demographical findings were followed by post-hoc analyses to compare the three groups (Bonferroni corrected).

First, we used a linear approach by applying multivariate regression models to evaluate the association between cumulative childhood trauma severity (CTQ-SF total score) and total gray matter volume within the total sample, followed by the individual (sub)cortical lobes. All models were adjusted for group (dummy coding healthy controls, bipolar type-I and schizophrenia-spectrum disorder) and the covariates age, sex, cerebral brain volume, medication status (binary variable representing whether or not the patient was currently on an antipsychotic, antidepressant or lithium) and type of scan (dummy variable, high-res T1 *v*. T1). Analyses were Hochberg-adjusted (Benjamini & Hochberg, 1995) for familywise multiple testing errors, using a false discovery rate (FDR) of 5% (*q* < 0.050). After identifying significant lobe(s), follow-up analyses were performed to specify which individual brain region(s) contributed to this effect (*p* < 0.050). Considering earlier sMRI findings focusing on the hippocampus and amygdala as single ROI's, we additionally defined these regions as specific outcomes of interest. Trauma-by-group interaction effects were examined by adding this interaction term in a separate set of analyses.

Second, a categorical approach was used to follow up on significant findings by means of the Jonckheere−Terpstra tests for ordered alternatives (J−T, one-tailed test) (Jonckheere, 1954). This, to address the general observations of low trauma scores in healthy control cohorts, in addition to a substantial number of patients not reporting any type of trauma. In the current sample, about 70% of the healthy control subgroup did not report any type of trauma according to CTQ cutoff-scores, in addition to more than 50% of both patient subgroups. For clinical relevance, individuals were first grouped according to the number of childhood trauma subtypes they had experienced using the established cut-off scores for moderate to severe trauma (Bernstein et al., 2003). This resulted in four categories: no trauma; one trauma subtype, two trauma subtypes; and three or more trauma subtypes (three, four or five trauma subtypes were combined due to the low number of participants in these categories). Moreover, to address potential concerns on the use of predefined cut-off values, we created quartiles based on the total sample distribution of cumulative childhood trauma scores. After splitting the total sample by subgroup, J-T tests were repeated for the bipolar-I patients, schizophrenia-spectrum patients and healthy controls separately.

## Results

### Demographic characteristics

**Fig. 1.**
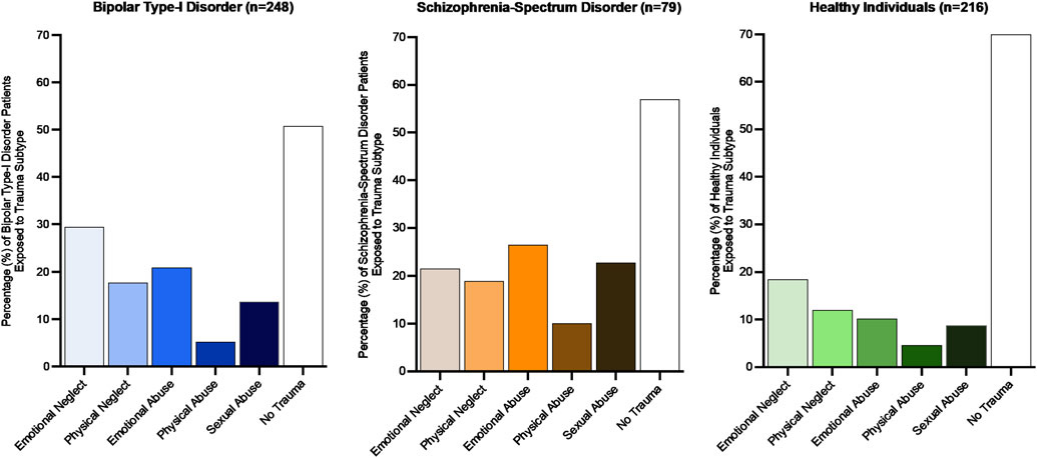
Number of individuals (%) exposed to different childhood trauma subtypes across the three subgroups.

The subgroup of schizophrenia-spectrum disorder patients (*n* = 79) was on average younger and included more male participants than the other subgroups (Table 1). Healthy controls (*n* = 216) were significantly younger than bipolar patients (*n* = 248). Mean childhood trauma severity (CTQ-total score) was significantly higher for both patient groups as compared to healthy controls (Table 1). Figure 1 displays the prevalence of the five childhood trauma subtypes across the three subgroups. The number of individuals reporting to at least one form of trauma was similar in the bipolar patients (49.2%) and the schizophrenia-spectrum group (43%), this percentage was significantly lower in the healthy control group (29.6%). Total cerebral brain volume was significantly smaller in the bipolar and schizophrenia-spectrum subgroups, as compared to controls (Table 1).

**Table 1.** Demographic characteristics of the included participants

	Bipolar-I patients (*n* = 248)	Schizophrenia- spectrum patients (*n* = 79)	Healthy controls (*n* = 216)	Test statistic		
Age, mean (s.d.), year	48.06 (12.20)	32.63 (11.16)	43.16 (14.53)	***F*(2) = 42.44**	***p* = 0.000**	BP > HC > SSP
Male (%)	48.0%	65.8%	44.9%	**χ^2^ = 10.47**	***p* = 0.005**	HC = BP > SSP
Childhood trauma						
CTQ total score, mean (s.d.)	40.47 (12.13)	43.06 (16.15)	35.37 (10.59)	***F*(2) = 15.59**	***p* < 0.001**	HC < BP = SSP
Childhood trauma (%)^a^	49.2%	43.0%	29.6%	**χ^2^ = 18.58**	***p* < 0.001**	HC < BP = SSP
MRI						
Total cerebral volume, mean (s.d.)	1 041 459.64 (100 808.56)	1 078 368.60 (111 656.83)	1 072 278.07 (107 554.89)	***F*(2) = 7.69**	***p* *=* 0.001**	HC > BP = SSP

s.d., standard deviation; CTQ, Childhood Trauma Questionnaire; BP, Bipolar-I patients; SSP, Schizophrenia-Spectrum Patients; HC, healthy controls.

a Individuals were categorized as having a history of childhood trauma when scoring above the cut-off for one or more trauma subtype(s). Bold values should be *p* < 0.001.

### Linear trauma-related gray matter volume alterations across the brain

Considering the total sample (*n* = 512), the negative association between childhood trauma severity and total gray matter volume was not significant (*β* = −0.021, *p* = 0.124), correcting for group (*p* = 0.361) and including age, sex, cerebral brain volume, medication status and type of scan. Childhood trauma was significantly related to reduced gray matter volume of the frontal lobe (*β* = −0.049, *p* = 0.008; FDR-corrected, *q* = 0.048, scatter plot provided in eFigure 1 in Supplement), but not to the volume of other (sub)cortical lobes (Table 2). Childhood trauma severity was not associated with gray matter volume of the hippocampus (left: *β* = 0.005, *p* = 0.883; right: *β* = −0.009, *p* = 0.802) or amygdala (left: *β* = 0.002, *p* = 0.953; right: *β* = −0.054, *p* = 0.133). Analyses did not show any significant group effects on gray matter volume and there was no evidence for trauma-by-group interactions in a separate set of analyses.

**Table 2.** Linear associations between childhood trauma severity and gray matter volumes

	*b*^a^	(s.e.)	*β*^a^	*p* Value	FDR-corrected *Q* value
Frontal lobe	**−65.17**	**(24.79)**	**−0.049**	**0.008**	**0.048**
Temporal lobe	−29.43	(16.47)	−0.037	0.075	0.225
Cingulate/Insular lobe	−11.90	(7.52)	−0.039	0.114	0.228
Subcortical lobe	−2.84	(8.74)	−0.007	0.746	0.943
Parietal lobe	−1.97	(17.36)	−0.002	0.910	0.943
Occipital lobe	0.91	(12.69)	0.002	0.943	0.943

s.e., standard error; FDR, False-Discovery Rate.

a Group, age, sex, cerebral brain volume, type of scan (high-resolution T1 *v*. T1), and medication status were included as covariates. Bold values should be *p* < 0.001.

Sensitivity analyses showed that the trauma-related gray matter volume reductions within the frontal lobe were localized in the right medialorbitofrontal region (*β* = −0.105, *p* = 0.002), right paracentral region (*β* = −0.088, *p* = 0.018), right superior frontal area (*β* = −0.058, *p* = 0.027) and left precentral region (*β* = −0.057, *p* = 0.0498) (eTable 1 in Supplement). Group had a significant effect on the left caudal middle frontal region (*β* = 0.129, *p* = 0.022) and bilateral rostral middle frontal regions (left: *β* = 0.143, *p* = 0.002; right: *β* = 0.087, *p* = 0.049). Post-hoc testing indicated larger gray matter volumes in the bipolar subgroup *v.* healthy controls (*p* = 0.013, *p* = 0.002 and *p* = 0.025, respectively). No trauma-by-group effects were observed.

### Categorical trauma-related gray matter alterations in the frontal lobe

**Fig. 2.**
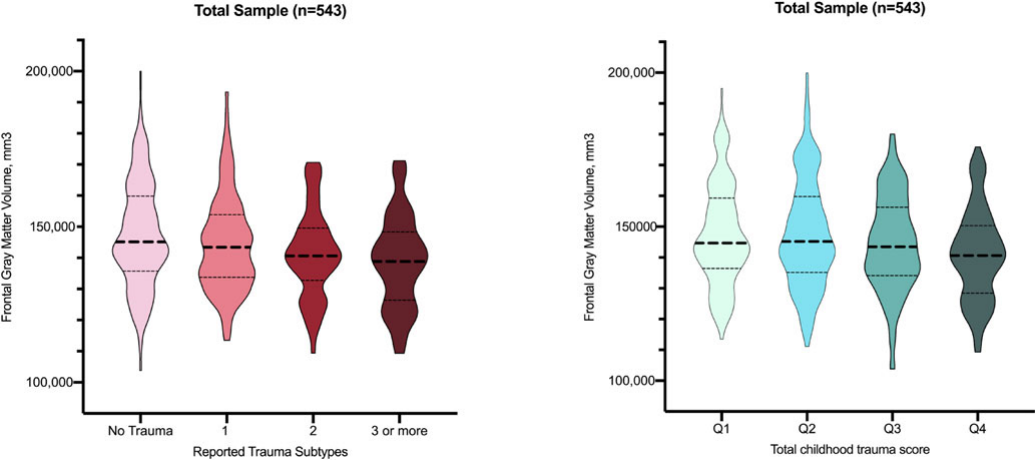
Violin plots depicting frontal gray matter volume across categories of reported trauma subtypes and cumulative trauma score in the total sample.

**Fig. 3.**
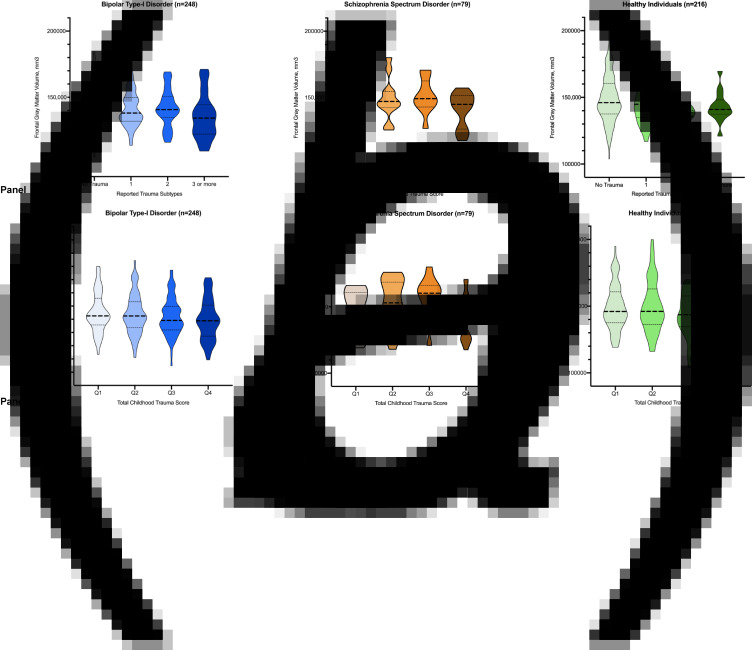
Violin plots depicting frontal gray matter volume across categories of reported trauma subtypes (*a*) and cumulative trauma score (*b*) within the three subgroups.

Including the total sample, more pronounced frontal gray matter volume reductions were found in individuals reporting an increasing number of childhood trauma subtypes (J-T = −3.86, *p* < 0.001, eTable 2, Fig. 2). Cumulative trauma scores, independent from trauma subtype, revealed a similar dose-response pattern: higher quartiles of overall trauma rates were linked to more pronounced gray matter volume reductions in the frontal lobe (J-T = −3.25, *p* = 0.001, eTable 2, Fig. 2). When repeating these analyses for the individual subgroups, similar significant dose-response patterns were observed in the bipolar-I patients and healthy controls, but did not reach significance for the schizophrenia-spectrum subgroup (eTable 2, Fig. 3).

## Discussion

Exploring both cortical and subcortical brain regions in the largest transdiagnostic sample to date, we found that childhood trauma was associated with reduced gray matter volume in the frontal lobe, while no trauma-related volume reductions were observed in the hippocampus or amygdala. Sensitivity analyses showed that the right medial orbitofrontal, paracentral, superior frontal regions and the left precentral region specifically contributed to this effect. These frontal trauma-related reductions may be shared across diagnostic groups, as no trauma-by-group interaction effects were present. Ranked-analyses confirmed more pronounced frontal gray matter reduction in those reporting multiple forms of childhood trauma and across quartiles of cumulative trauma severity. Similar dose-response patterns were also significant within the subgroups of bipolar disorder patients and healthy controls.

The suggestion that the frontal regions are particularly sensitive to childhood trauma is supported by animal studies (Schubert, Porkess, & Dashdorj, 2009), showing structural changes in prefrontal dendrites after 1 week of induced stress (e.g. maternal separation or isolation rearing), or even a single traumatic incident, while changes in the hippocampus are observed only after enduring stress for several weeks (Arnsten, 2009). Studies in maltreated children and adolescent samples have consistently shown frontal gray matter reductions, rather than hippocampal or amygdala reductions (De Brito et al., 2013; Edmiston et al., 2011; Hanson et al., 2010; Kelly et al., 2013). Results from prior adult whole-brain studies in smaller samples, including patients as well as healthy subjects, also support a dose-response effect of trauma on the frontal lobe (Lim et al., 2014; Paquola et al., 2016). Moreover, an ENIGMA study with the largest multi-center sample to date (*n* = 3036, healthy controls and patients with major depressive disorder) (Frodl et al., 2017) investigated the link between childhood adversity and subcortical brain volume and did not replicate previous reports on trauma-related hippocampal or amygdala reductions either (Lim et al., 2014; Paquola et al., 2016).

Although brain functioning related to cognition and social-emotional behavior is complex and distributed across remote brain regions, the frontal lobe is generally implicated to support top-down regulations of affect, motivational processing and social-emotional behaviors as well as higher-order cognition (Bonelli & Cummings, 2007; Carrion & Wong, 2012; Dolan, 2007; Lim et al., 2014; Menon, 2011). Trauma-related cognitive and affective deficits may be mediated by structural brain alterations, predominantly in the frontal cortex. A link between early maltreatment and affective problems as well as cognitive deficits has consistently been shown in patient samples and healthy cohorts (Barzilay et al., 2019; Gould et al., 2012; McCrory, De Brito, & Viding, 2011; Pechtel & Pizzagalli, 2011; Wilson, Hansen, & Li, 2011). Our group previously reported that higher trauma severity is associated with reduced adaptive stress reactivity (Begemann, Stotijn, Schutte, Heringa, & Sommer, 2017), high levels of neuroticism (So, Begemann, Gong, & Sommer, 2016), and lower inhibitory control (Begemann, Daalman, Heringa, Schutte, & Sommer, 2016; Begemann, Heringa, & Sommer, 2016). Trauma-susceptible brain regions can be particularly impacted by adverse events during so called ‘sensitive periods’ (Anda et al., 2006; Sánchez et al., 2001). Notably, the frontal lobe matures relatively late and its protracted development could make this region vulnerable to negative environmental influences for a longer time than other regions with more rapid maturation. Suboptimal functioning may not become apparent until full brain maturation in late adolescence or early adulthood, which is the time when bipolar disorder and schizophrenia-spectrum disorder are typically diagnosed (Bachevalier & Loveland, 2006).

An important finding of the current study is that these trauma-related frontal gray matter reductions were observed in a transdiagnostic sample of healthy individuals, bipolar disorder patients and schizophrenia-spectrum disorder patients, without significant trauma-by-group interactions. This could indicate that trauma-related reductions in the frontal (sub)regions are shared across psychiatric patients, independent of diagnosis. In healthy individuals, the neurobiological effects of trauma have been described as being more subtle (Souza-Queiroz et al., 2016), with lower trauma rates and smaller variance compared to patient cohorts, requiring large samples to demonstrate these effects. Both our ranked analyses revealed a clear pattern of more pronounced frontal gray matter reduction in healthy individuals reporting multiple forms of childhood trauma and across quartiles of cumulative trauma severity. While few studies that investigated healthy subjects extended their search beyond the hippocampal and amygdala regions, some previous studies have reported trauma-related frontal gray matter reductions in healthy adults (*n* = 145) and healthy adolescents (*n* = 42) (Dannlowski et al., 2012; Edmiston et al., 2011).

The present data provide strong evidence that childhood trauma contributes to the neurobiological changes commonly observed in psychiatric disorders. The categorical dose-response patterns were clearly observed in the bipolar disorder patients. In the schizophrenia-spectrum disorder subgroup, an identical association was found between more pronounced frontal gray matter reductions in individuals reporting multiple forms of trauma, with a more subtle pattern for quartiles of overall trauma rates – yet our subgroup of 79 patients appeared underpowered to demonstrate these associations (May & Looney, 2020). Previous neuroimaging studies have shown frontal gray matter alterations in bipolar disorder (Duarte et al., 2016; Souza-Queiroz et al., 2016), schizophrenia-spectrum disorder (Hoy et al., 2012; Sheffield, Williams, Woodward, & Heckers, 2013), major depression disorder (Lu et al., 2019; Vythilingam et al., 2002), posttraumatic stress disorder (O'Doherty et al., 2017), borderline personality disorder (Brunner et al., 2010), and substance use disorder (Bachi et al., 2018; Van Dam, Rando, Potenza, Tuit, & Sinha, 2014). An overlap in frontal gray matter loss has been demonstrated when directly comparing patients with bipolar disorder and those with a schizophrenia-spectrum disorder (Arnone et al., 2009), but also when evaluating bipolar disorder *v.* depression (Wise et al., 2017), and depression *v.* borderline personality disorder (Depping et al., 2016). Thus, the so-called *p*-factor (Caspi et al., 2014; Selzam, Coleman, Caspi, Moffitt, & Plomin, 2018), reflecting the general effect of having a psychiatric illness including shared structural brain abnormalities across disorders (Opel et al., 2020; Parker et al., 2020), may overlap (perhaps for a large part) with the ‘t-factor’ reflecting the independent impact of childhood trauma.

### Strengths and limitations

The current study has notable strengths, including its transdiagnostic design and extending the search for trauma-related gray matter alterations in brain regions other than the hippocampus and amygdala. Ideally, we would have implemented a fully data-driven approach based on individual brain regions. To ensure statistical power, we chose to conduct a lobe-wise analysis and follow up on significant results with explorative and uncorrected analyses to examine which individual regions would drive a particular result. Moreover, the three groups differed in age and gender distribution, in part because these are disease specific variables. Given the absence of trauma-by-group interactions in the total sample and the fact that we found a similar association between trauma and frontal brain volume across two out of three subgroups, suggests that age and gender effects do not to limit the interpretation of our current main findings. Moreover, we could not account for the plausible effect of cumulative medication use on gray matter volume, but instead evaluated current medication status. We also note that two different structured interviews were used to confirm clinical diagnosis in all included patients (SCID and CASH), their treating physician had already established diagnosis prior to enrollment. Furthermore, responses to the retrospective self-report questionnaire measuring childhood trauma may have been influenced by the inability of respondents to remember such experiences and subjective perception of the exposure to trauma. However, the CTQ-SF is currently indicated as one of the most reliable instruments for evaluating childhood trauma in healthy individuals as well as patients and our found trauma rates are in line with previous reports (Álvarez et al., 2011; Bernstein et al., 2003; Devi et al., 2019; Schäfer & Fisher, 2011; Wiersma et al., 2009). Notably, the timing of maltreatment during childhood is not specifically assessed by the CTQ. The neurobiological effects on the brain, particularly limbic structures (Herzog et al., 2020), may depend on the timing of these experiences, which could be relevant for our null-findings regarding limbic structures. Important goals for future work should include setting up longitudinal cohort studies and analyzing large-scale transdiagnostic international consortium data sets.

In conclusion, our findings indicate that the frontal regions are most sensitive to the impact of childhood trauma and that these trauma-related frontal reductions are independent from psychiatric comorbidity. The frontal areas develop relatively late and the long window of high neuroplasticity may render these areas more vulnerable to adverse environmental circumstances during development. Emotional and cognitive deficits seen in individuals with a traumatic youth across diagnoses may at least partly stem from these frontal volume alterations and contribute to the development of psychopathology.

## Supplementary material

For supplementary material accompanying this paper visit https://doi.org/10.1017/S0033291721002087. click here to view supplementary material

## Trial registration

The study *Simvastatin for recent-onset psychosis* is registered in ClinicalTrails.gov (NCT01999309) and EudraCT (2013-000834-36).
