# Supplementary material for: Childhood trauma is associated with reduced frontal gray matter volume: a large transdiagnostic structural MRI study
Source: Psychol Med. 2021 Jun 3;53(3):741–9. doi: 10.1017/S0033291721002087 (PMC9975993; doi:10.1017/S0033291721002087)
Supplement: Supplementary file 1 [file S0033291721002087sup.zip › S0033291721002087sup001.docx]

**eAppendix**

**Methods**

A total of 554 subjects were recruited as part of three different studies, conducted in the University Medical Center Utrecht (the Netherlands): *Bipolar Genetics study*, *Spectrum* *study* and *Simvastatin for recent-onset psychosis.* All participants were ≥18 years of age. Healthy control subjects did not have a current or past psychiatric diagnosis. Patients met Diagnostic and Statistical Manual for Mental Disorders, Fourth Edition (DSM-IV; American Psychiatric Association, 2000) criteria for bipolar disorder type-I or a schizophrenia spectrum disorder. Studies were approved by the relevant medical ethical committee, all participants gave written informed consent.

The *Bipolar Genetics study* investigated genetic and phenotypic information of patients with bipolar disorder type-I, first-degree relatives and controls (Vreeker et al., 2015). For the current study, we included 250 bipolar type-I patients who were recruited via clinicians, the Dutch patients’ association, pharmacies and advertisements. In addition, we included 133 controls that were enrolled via advertisements and by inviting individuals who previously participated in a study and had agreed to be contacted for new research. Presence or absence of psychopathology was established using the Structured Clinical Interview for the Diagnostic and Statistical Manual of Mental Disorders DSM-IV (SCID-I, First et al. 1997).

The *Spectrum* *study* examined psychotic experiences across the continuum of patients with a psychotic disorder and the general population. We included data from 47 patients with a schizophrenia spectrum disorder (schizophrenia=29, psychosis not otherwise specified=18) who were enrolled via clinicians and the Dutch patients’ association. Healthy controls were recruited by means of a website ‘www.verkenuwgeest.nl’ (‘explore your mind’, see Sommer et al. 2010). Visitors of this Website filled out a self-report questionnaire that is designed to quantify the tendency to hallucinate in healthy individuals (modified version of the Launay and Slade Hallucination Scale [LSHS]), they were subsequently invited to visit the UMCU. Psychiatric diagnosis (in patients) or the absence of psychopathology (in healthy individuals) was evaluated using the Comprehensive Assessment of Symptoms and History Interview (CASH, Andreasen 1992). Of the 87 healthy individuals included in the current study, 37 reported auditory verbal hallucinations (at least once a month, for more than 1 year). These individuals are regarded as healthy controls since the presence of a psychotic disorder had been ruled out by a psychiatrist, as they did not show professional, psychological or social dysfunction. We know that these individuals report similar high rates of childhood trauma as found in patients (Begemann et al., 2017), thereby inclusion leads to a better distribution of childhood trauma rates in the healthy control group.

*Simvastatin for recent-onset psychosis* was a medication trial (Begemann et al. 2016), investigating the augmenting effects of simvastatin treatment (40 mg/daily) versus placebo. Participants used study medication for one year and visited the UMCU approximately once every three months for study measurements. We included baseline data for 37 (schizophrenia=16, delusional disorder=1, psychosis not otherwise specified=20) patients with a diagnosis of a schizophrenia spectrum disorder, who had been recruited via collaborating health institutions. Psychiatric diagnosis was confirmed using the Comprehensive Assessment of Symptoms and History Interview (CASH, Andreasen 1992).
