# Supplementary material for: Childhood trauma is associated with reduced frontal gray matter volume: a large transdiagnostic structural MRI study
Source: Psychol Med. 2021 Jun 3;53(3):741–9. doi: 10.1017/S0033291721002087 (PMC9975993; doi:10.1017/S0033291721002087)
Supplement: Supplementary file 1 [file S0033291721002087sup.zip › S0033291721002087sup002.docx]

**eTable 1**: Associations Between Childhood Trauma Severity and Gray Matter Volume of Individual Frontal Subregions.

| **Childhood trauma severity  as a predictor for GMV** | **Beta^a^** | **P value** | **Beta^a^** | **P value** |
| --- | --- | --- | --- | --- |
|  | Left hemisphere | | Right hemisphere | |
| Caudal middle frontal | -0.021 | .544 | -0.034 | .356 |
| Frontal pole | -0.003 | .949 | -0.031 | .453 |
| Lateral orbitofrontal | -0.045 | .127 | -0.059 | .057 |
| Medial orbitofrontal | -0.026 | .456 | **-0.105** | **.002** |
| Paracentral | -0.053 | .178 | **-0.088** | **.018** |
| Pars opercularis | -0.051 | .151 | -0.031 | .397 |
| Pars orbitalis | -0.066 | .053 | -0.042 | .254 |
| Pars triangularis | -0.022 | .546 | -0.020 | .596 |
| Precentral | **-0.057** | **.0498** | -0.052 | .079 |
| Rostral middle frontal | -0.014 | .612 | 0.003 | .920 |
| Superior frontal | -0.039 | .129 | **-0.058** | **.027** |

*Note*. GMV, gray matter volume. ^a^Group, age, sex, cerebral brain volume, type of scan (high-resolution T1 vs T1), and medication status were included as covariates. Significant findings are indicated in **bold**.
