# Supplementary material for: Childhood trauma is associated with reduced frontal gray matter volume: a large transdiagnostic structural MRI study
Source: Psychol Med. 2021 Jun 3;53(3):741–9. doi: 10.1017/S0033291721002087 (PMC9975993; doi:10.1017/S0033291721002087)
Supplement: Supplementary file 1 [file S0033291721002087sup.zip › S0033291721002087sup004.docx]

**eTable 2.** Associations Between Frontal Gray Matter Volume and Categories of Reported Trauma Subtypes and Cumulative Trauma Score in the Total Sample and Subgroups.

|  | **Total Sample  (n = 543)** | | **Bipolar Type-I Disorder**  **(n = 248)** | | **Schizophrenia-Spectrum disorder  ( n= 79)** | | **Healthy Controls**  **(n = 216)** | |
| --- | --- | --- | --- | --- | --- | --- | --- | --- |
| **Ranking according to trauma subtypes** |  | |  | |  | |  | |
| No trauma | n = 323 | | n = 126 | | n = 45 | | n = 152 | |
| 1 trauma subtype | n = 109 | | n = 62 | | n = 11 | | n = 36 | |
| 2 trauma subtypes | n = 59 | | n = 35 | | n = 10 | | n = 14 | |
| ≧3 trauma subtypes | n = 52 | | n = 25 | | n = 13 | | n = 14 | |
| Jonckheere-Terpstra, Kendall’s tau | **Std. J-T=-3.86** (**p=.001**) **τb=-.13** |  | **Std. J-T=-2.47 (p=.013) τb=-.12** |  | Std. J-T=-1.55 (p=.122) τb=-.14 |  | **Std. J-T=-2.51 (p=.012) τb=-.14** |  |
|  |  | |  | |  | |  | |
| **Ranking according to cumulative trauma scores (quartiles)** |  | |  | |  | |  | |
| Q1 | n = 156 | | n = 51 | | n = 16 | | n = 89 | |
| Q2 | n = 135 | | n = 57 | | n = 22 | | n = 56 | |
| Q3 | n = 117 | | n = 66 | | n = 13 | | n = 38 | |
| Q4 | n = 135 | | n = 74 | | n = 28 | | n = 33 | |
| Jonckheere-Terpstra, Kendall’s tau | **Std. J-T=-3.25 (p=.001) τb=-.10** |  | **Std. J-T=-2.00 (p=.047) τb=-.10** |  | Std. J-T=-0.78 (p=.432) τb=-.07 |  | **Std. J-T=-2.32 (p=.021) τb=-.12** |  |

Abbreviations: J-T, Jonckheere-Terpstra; τb, Kendall’s Tau-b (as effect size estimate). Significant findings in **bold**.
