# Supplementary figures and images for: Childhood trauma is associated with reduced frontal gray matter volume: a large transdiagnostic structural MRI study
Source: Psychol Med. 2021 Jun 3;53(3):741–9. doi: 10.1017/S0033291721002087 (PMC9975993; doi:10.1017/S0033291721002087)

## Slide 1
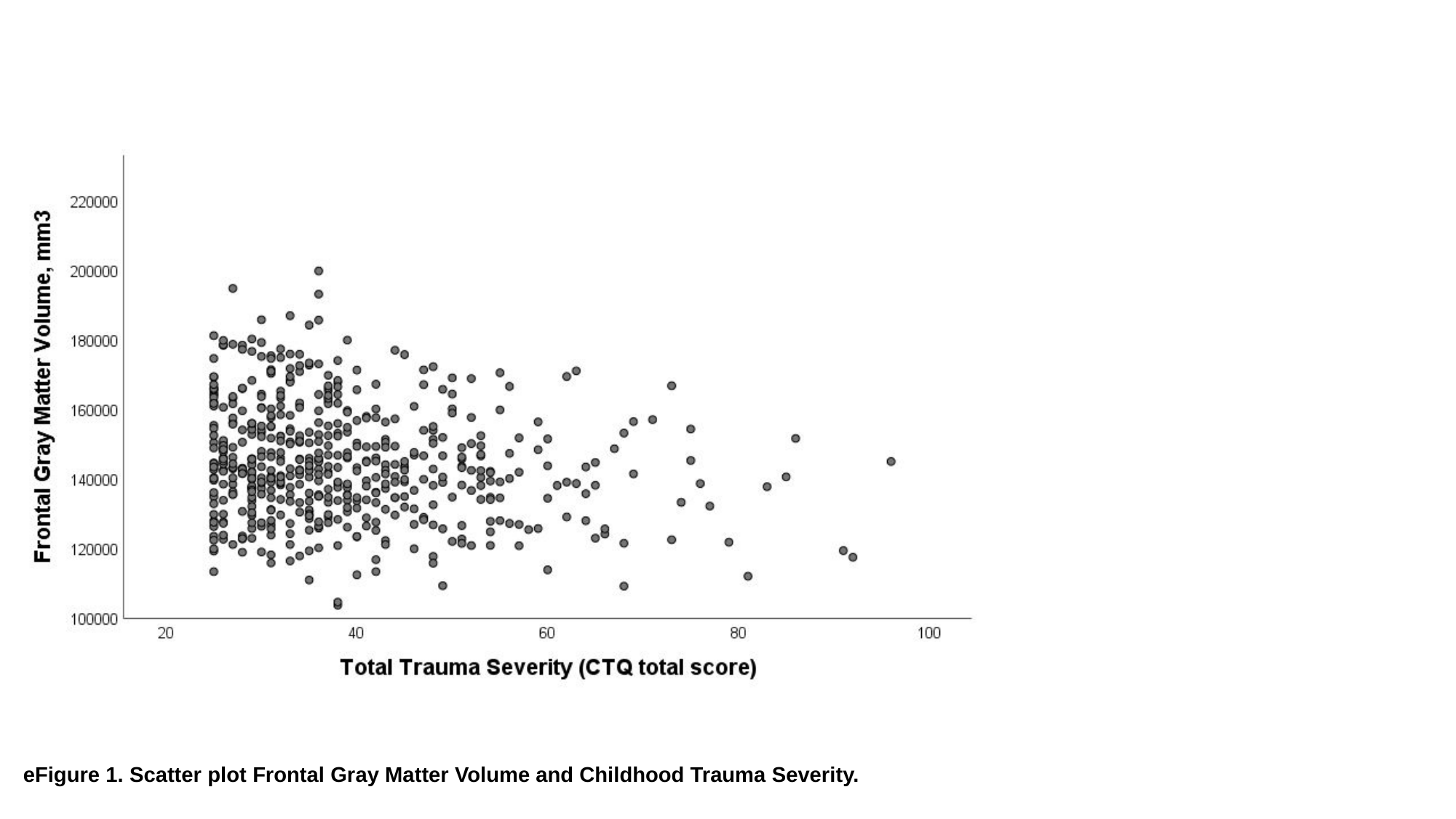

eFigure 1. Scatter plot Frontal Gray Matter Volume and Childhood Trauma Severity.

Supplement: Supplementary file 1 [file S0033291721002087sup.zip › S0033291721002087sup003.pptx]
